# Stress-induced hemostasis: mechanisms and implications for health

**DOI:** 10.1097/MS9.0000000000003012

**Published:** 2025-05-21

**Authors:** Emmanuel Ifeanyi Obeagu

**Affiliations:** Department of Biomedical and Laboratory Science, Africa University, Mutare, Zimbabwe

**Keywords:** coagulation, health implications, hemostasis, inflammatory pathways, stress

## Abstract

Stress, encompassing both psychological and physical dimensions, profoundly affects the hemostatic system, which is responsible for maintaining the delicate balance between blood fluidity and clot formation. The interplay between stress and hemostasis involves intricate mechanisms where stress hormones like cortisol, adrenaline, and noradrenaline interact with coagulation factors and platelets, promoting a hypercoagulable state. This review delves into the various pathways through which stress influences hemostasis, highlighting the critical roles of the sympathetic nervous system, the hypothalamic-pituitary-adrenal axis, and inflammatory mediators. Psychological stress, arising from anxiety, depression, and acute mental stress, has been shown to elevate levels of coagulation factors such as fibrinogen and D-dimer, increasing the risk of thrombotic events. Similarly, physical stress from intense exercise or trauma enhances platelet reactivity and the expression of procoagulant factors. The body’s response to severe physical stress, such as trauma or burns, can overwhelm the hemostatic system, leading to conditions like disseminated intravascular coagulation, characterized by widespread clotting and subsequent bleeding. The health implications of stress-induced hemostatic changes are significant, particularly concerning cardiovascular diseases. Chronic stress contributes to persistent hypercoagulability, elevating the risk of myocardial infarction and stroke. Acute stress episodes can precipitate acute coronary syndromes, especially in individuals with preexisting conditions like hypertension or diabetes.

## Introduction

Stress, a ubiquitous aspect of modern life, exerts profound effects on both psychological and physiological systems^[1]^. It is well-documented that stress can trigger a cascade of biological responses, impacting various bodily functions, including the cardiovascular, immune, and endocrine systems. One critical system that is significantly influenced by stress is the hemostatic system, which plays a crucial role in maintaining blood fluidity and preventing excessive bleeding through the process of blood clotting. Hemostasis is a complex physiological process involving the interplay of blood vessels, platelets, and coagulation factors, all of which must work in harmony to prevent both hemorrhage and pathological clot formation. The body’s hemostatic balance can be disrupted by various forms of stress, leading to either a hypercoagulable state, which increases the risk of thrombosis, or a hypocoagulable state, which can result in excessive bleeding^[2]^. The response to stress involves the activation of the hypothalamic-pituitary-adrenal (HPA) axis and the sympathetic nervous system (SNS), both of which release a variety of hormones and neurotransmitters^[3]^. These biochemical messengers, including cortisol, adrenaline, and noradrenaline, have potent effects on the hemostatic system. For instance, adrenaline and noradrenaline can enhance platelet aggregation and activate coagulation pathways, while cortisol can influence the synthesis of coagulation factors and modulate inflammatory responses, further impacting hemostatic balance. Psychological stress, encompassing experiences such as anxiety, depression, and acute mental stress, has been extensively studied for its impact on hemostasis. Research indicates that individuals under psychological stress exhibit elevated levels of coagulation factors like fibrinogen and von Willebrand factor (vWF), which are associated with an increased risk of thrombotic events such as myocardial infarction and stroke^[4]^. Furthermore, psychological stress can alter platelet function, making them more prone to aggregation and contributing to a pro-thrombotic state.

**Highlights**

- Sympathetic nervous system: Stress activates catecholamines, enhancing platelet activation, coagulation, and vasoconstriction, increasing clot formation risk.
- HPA axis activation: Cortisol boosts clotting factors and inhibits fibrinolysis, contributing to a procoagulant state.
- Inflammatory response: Stress-induced inflammation increases cytokines and endothelial dysfunction, promoting blood clot formation and cardiovascular risks.
- Oxidative stress: ROS from stress enhance platelet activation and coagulation, while damaging endothelial cells and promoting thrombosis.
- Hormonal effects: Stress alters hormones like cortisol and adrenaline, influencing platelet function, coagulation factors, and vascular responses.

Physical stress, including intense exercise, trauma, and surgery, similarly affects hemostatic mechanisms. During physical stress, the body’s immediate response includes the release of stress hormones and the activation of the coagulation cascade to prepare for potential injury and blood loss^[5]^. While moderate exercise can have beneficial effects on cardiovascular health, intense and prolonged physical stress can enhance platelet reactivity and increase the levels of procoagulant factors, heightening the risk of thrombosis. The interplay between stress and hemostasis is mediated through several pathways^[6]^. The activation of the SNS leads to the release of catecholamines, which directly influence platelet function and vascular tone. The HPA axis, through the release of glucocorticoids like cortisol, modulates the production of clotting factors and the sensitivity of platelets to activation. Additionally, stress-induced inflammatory responses contribute to the upregulation of cytokines such as interleukin-6 (IL-6) and tumor necrosis factor-alpha (TNF-α), which can further influence coagulation processes. The implications of stress-induced hemostatic changes for health are vast. Chronic stress, characterized by sustained elevations in stress hormones and prolonged activation of hemostatic pathways, is a known risk factor for cardiovascular diseases^[7]^. Chronic hypercoagulability can lead to the formation of atherosclerotic plaques and increase the likelihood of thrombotic events. Acute stress episodes, such as those experienced during natural disasters or severe emotional distress, can precipitate acute coronary syndromes and other cardiovascular emergencies. Individuals with preexisting conditions such as hypertension, diabetes, and obesity are particularly susceptible to the adverse effects of stress on hemostasis. These conditions often involve baseline alterations in hemostatic balance, which can be exacerbated by stress, leading to an increased risk of thrombotic complications. Therefore, understanding how stress affects hemostasis in these populations is essential for developing targeted interventions to reduce cardiovascular risk. In light of these considerations, it is crucial to explore therapeutic strategies that can mitigate the impact of stress on hemostasis. Stress management techniques, such as mindfulness-based stress reduction, cognitive-behavioral therapy, and physical exercise, have shown promise in reducing stress levels and improving hemostatic balance. Pharmacological interventions, including the use of anticoagulants and anti-inflammatory agents, may also play a role in managing stress-induced hypercoagulability, particularly in high-risk populations.

## Aim

The aim of this review is to comprehensively explore the mechanisms and health implications of stress-induced hemostatic changes.

## Rationale

In contemporary society, where both psychological and physical stressors are ubiquitous, there is an urgent need to explore how these stressors influence the body’s hemostatic system—the intricate network responsible for regulating blood clotting and maintaining vascular integrity. The rationale for this review stems from several key observations and emerging concerns that underscore the significance of this topic. The prevalence of stress in modern life is undeniable. People face a constant barrage of stressors, from work pressures and academic demands to personal challenges and global uncertainties. This high-stress environment is a growing public health concern, as chronic stress is linked to numerous adverse health outcomes. Yet, despite this recognition, there remains a gap in understanding the specific physiological mechanisms through which stress affects hemostasis. While stress is widely acknowledged to contribute to conditions such as cardiovascular diseases, there is a need for a detailed exploration of how stress-induced changes in the hemostatic system contribute to these outcomes. Recent advancements in stress research have revealed that stress affects more than just psychological well-being. It influences biological systems in ways that are not always immediately visible but have long-term health consequences. Stress triggers a cascade of physiological responses, including hormonal changes, inflammatory processes, and alterations in vascular function. These responses can significantly impact hemostatic balance, either promoting excessive clotting or contributing to bleeding disorders.

The complex interplay between stress and hemostasis is increasingly recognized as a key factor in the development of chronic diseases. Research has shown that stress can exacerbate cardiovascular conditions through mechanisms such as increased platelet activation, enhanced coagulation, and inflammatory responses. However, there is still a need for a comprehensive review that integrates these findings to provide a clearer picture of how stress-induced hemostatic changes contribute to the onset and progression of diseases like atherosclerosis, venous thromboembolism (VTE), and hypertension. Such a review would help clarify how these processes interact and identify potential points for intervention. There is a growing interest in personalized medicine and the search for tailored approaches to health management. By exploring the links between stress, hemostatic balance, and specific health outcomes, this review aims to highlight opportunities for individualized treatment plans and preventive measures that address both the physiological and psychological aspects of stress. The need for practical management strategies to mitigate the effects of stress on health is more pressing than ever. While various approaches to stress management exist, there is a need for a synthesized review that not only summarizes current knowledge but also explores new avenues for effective stress management. This review aims to bridge the gap between research and practice by discussing current therapeutic strategies, such as lifestyle modifications and pharmacological interventions, and suggesting future directions for research and clinical practice.

## Review methodology

### Literature search and selection

The next phase involved a rigorous and systematic search for relevant literature. The search strategy included several key components:

1. Database selection: A variety of academic databases were utilized to ensure a broad and inclusive search. These databases included PubMed, Scopus, Web of Science, PsycINFO, and Google Scholar. These sources were chosen for their comprehensive coverage of biomedical, psychological, and clinical research.
2. Search terms and keywords: A set of well-defined search terms and keywords was developed to capture relevant studies. Terms included “stress,” “hemostasis,” “blood clotting,” “psychological stress,” “physical stress,” “coagulation,” “inflammation,” “cardiovascular disease,” “autoimmune disorders,” “metabolic syndrome,” and “stress management.” Boolean operators (AND, OR) were used to combine these terms effectively.
3. Inclusion and exclusion criteria: Criteria for selecting studies were established to ensure that only high-quality and relevant research was included. Inclusion criteria included:
  - Studies published in peer-reviewed journals.
  - Research focusing on stress and its effects on hemostasis.
  - Articles addressing both psychological and physical stressors.
  - Studies that explore health implications and management strategies.

Exclusion criteria included:

- Studies not focused on human subjects.
- Articles not published in English.
- Research that did not address stress or hemostasis directly.

4. Screening process: The initial search yielded a large number of articles. A two-step screening process was used to narrow the selection. First, titles and abstracts were reviewed for relevance. Second, full-text articles were assessed for alignment with the review’s scope and objectives. This process involved careful evaluation to exclude studies that did not meet the inclusion criteria or were of insufficient methodological quality.

### Hemostasis and its phases

Hemostasis is a critical physiological process that ensures the stoppage of bleeding and maintains the integrity of the circulatory system^[8]^. This complex mechanism involves the coordinated action of blood vessels, platelets, and plasma proteins. Hemostasis is traditionally divided into three main phases: vascular spasm, platelet plug formation, and coagulation. Each phase is vital for preventing excessive blood loss and ensuring proper wound healing. The first response to vascular injury is vascular spasm, also known as vasoconstriction. This immediate reflex constriction of the blood vessel is triggered by the direct injury to the vascular smooth muscle, the release of signaling molecules such as endothelin, and the reflexes from pain receptors. The primary function of vascular spasm is to reduce blood flow to the injured area, minimizing blood loss and creating a favorable environment for the subsequent hemostatic processes. This phase is short-lived but crucial as it provides the initial control of hemorrhage. Following vascular spasm, the second phase of hemostasis is the formation of a platelet plug. Platelets, or thrombocytes, play a central role in this process. When a blood vessel is damaged, the underlying collagen and other extracellular matrix components are exposed. Platelets adhere to these exposed structures through specific receptors, such as glycoprotein Ib, which binds to vWF anchored to the subendothelial matrix. This adhesion activates the platelets, causing them to change shape, release granule contents (such as ADP, serotonin, and thromboxane A2), and recruit additional platelets to the site of injury.

The recruited platelets aggregate, forming a temporary platelet plug those seals small vascular breaches. This aggregation is facilitated by fibrinogen bridges that link platelets through their glycoprotein IIb/IIIa receptors. The platelet plug provides an initial barrier to blood loss, but it is relatively weak and needs reinforcement from the coagulation cascade to ensure stability^[8]^. The third and final phase of hemostasis is coagulation, which involves a complex series of enzymatic reactions known as the coagulation cascade. This cascade consists of two initial pathways, the intrinsic and extrinsic pathways, which converge on a common pathway leading to the formation of a stable fibrin clot. This pathway is initiated by damage to the endothelium and exposure of blood to subendothelial collagen. It involves several coagulation factors (XII, XI, IX, and VIII) and is measured by the activated partial thromboplastin time in clinical settings. Triggered by external trauma leading to the exposure of tissue factor (TF), which is present on the surface of subendothelial cells. TF interacts with factor VII, and this interaction is crucial for the rapid initiation of coagulation. The extrinsic pathway is assessed by the prothrombin time test. Both pathways converge at the activation of factor X, which, along with factor V, calcium ions, and phospholipids, forms the prothrombinase complex. This complex converts prothrombin (factor II) into thrombin (factor IIa). Thrombin is a key enzyme that not only converts fibrinogen into fibrin but also activates factors V, VIII, and XIII, amplifying the coagulation cascade and stabilizing the fibrin clot. Factor XIIIa cross-links fibrin strands, creating a dense, stable network that secures the platelet plug and forms a definitive clot^[8]^. Hemostasis is tightly regulated to prevent excessive clotting (thrombosis) or insufficient clotting (hemorrhage)^[9]^. Several anticoagulant mechanisms exist, including the action of antithrombin, protein C and protein S pathways, and tissue factor pathway inhibitor. These regulatory pathways ensure that coagulation is limited to the site of vascular injury and that clot formation is balanced with clot dissolution (fibrinolysis), primarily mediated by plasmin.

### Stress and the hemostatic system

The hemostatic system, which maintains the delicate balance between blood clotting and fluidity, is highly susceptible to the effects of stress. This intricate system comprises a series of interrelated processes, including vascular constriction, platelet aggregation, and the activation of the coagulation cascade. Stress, whether psychological or physical, can significantly alter these processes, leading to a range of hemostatic changes with important health implications^[9]^. When an individual experiences stress, the body responds by activating the HPA axis and the SNS^[10]^. This activation results in the release of several key hormones, including cortisol, adrenaline, and noradrenaline. These stress hormones play a crucial role in preparing the body to deal with the stressor but also have significant effects on the hemostatic system. Cortisol, a glucocorticoid hormone released from the adrenal cortex, affects hemostasis in multiple ways^[11]^. It influences the synthesis and activity of various coagulation factors, such as factor VIII and vWF, and modulates the inflammatory response, which can impact clot formation. Elevated cortisol levels can lead to increased production of procoagulant factors, contributing to a hypercoagulable state.

Adrenaline and noradrenaline, catecholamines released from the adrenal medulla, enhance platelet aggregation and activation^[12]^. These hormones increase the expression of adhesion molecules on the surface of platelets, making them more likely to stick together and form clots. Additionally, catecholamines can induce vasoconstriction, reducing blood flow and promoting clot formation in small blood vessels. Psychological stress, stemming from factors such as anxiety, depression, and acute mental stress, has been shown to influence hemostatic parameters significantly. Studies indicate that individuals under psychological stress exhibit elevated levels of coagulation factors, including fibrinogen, factor VII, and D-dimer^[4]^. These changes promote a pro-thrombotic state, which can increase the risk of cardiovascular events. Chronic psychological stress is particularly concerning as it leads to sustained activation of the HPA axis and the SNS^[13]^. This prolonged activation can result in continuous elevation of stress hormones, leading to persistent hypercoagulability. Over time, this can contribute to the development of atherosclerosis and increase the risk of myocardial infarction and stroke. Physical stress, such as that resulting from intense exercise, trauma, or surgery, also impacts hemostasis^[14]^. During vigorous physical activity, the body’s immediate response includes the release of stress hormones, which enhance platelet reactivity and increase the levels of procoagulant factors. This response is part of the body’s preparation for potential injury, facilitating quick clot formation to prevent bleeding. In cases of severe physical stress, such as major trauma or burns, the hemostatic system can be overwhelmed. This can lead to disseminated intravascular coagulation (DIC), a condition characterized by widespread clotting and subsequent bleeding due to the depletion of clotting factors and platelets. DIC is a severe and often life-threatening condition that requires immediate medical intervention.

### Mechanisms of stress-induced hemostatic changes

Stress is a complex physiological and psychological state that profoundly affects the body’s hemostatic system, which is responsible for regulating blood clotting and maintaining vascular integrity. The mechanisms through which stress induces hemostatic changes are intricate, involving a range of biological systems and pathways. One of the primary mechanisms through which stress affects hemostasis is the activation of the SNS. Stress triggers the “fight-or-flight” response, a physiological reaction orchestrated by the SNS. This activation leads to the release of catecholamines, such as adrenaline and noradrenaline, from the adrenal medulla. These hormones have several effects on the hemostatic system: Catecholamines enhance platelet aggregation and activation, which increases the risk of blood clot formation. Adrenaline and noradrenaline bind to adrenergic receptors on platelets, leading to increased thromboxane A2 production, a molecule that promotes platelet activation and aggregation. Catecholamines also affect the coagulation cascade by increasing the levels of clotting factors. This is achieved through the stimulation of the liver to produce more coagulation factors such as factor VII, factor X, and fibrinogen, which promote blood clot formation. Stress-induced catecholamines cause vasoconstriction, which can increase blood pressure and enhance the conditions for clot formation by reducing blood flow and increasing shear stress on the vascular endothelium^[15]^.

Another key mechanism of stress-induced hemostatic changes is the activation of the HPA axis^[16]^. The HPA axis is a major regulatory system for the body’s response to stress, involving the release of cortisol, a steroid hormone produced by the adrenal cortex. Cortisol affects hemostasis in several ways: Cortisol increases the synthesis of procoagulant factors. It upregulates the production of clotting factors in the liver and enhances platelet aggregation, contributing to a hypercoagulable state. Cortisol inhibits fibrinolysis, the process that breaks down clots. It decreases the production of tissue plasminogen activator (tPA) and increases the production of plasminogen activator inhibitor-1, both of which inhibit the dissolution of blood clots. Cortisol has complex effects on inflammation, which can indirectly influence hemostasis. While cortisol generally has anti-inflammatory effects, chronic stress leads to sustained high levels of cortisol, which can perpetuate inflammatory states that affect the hemostatic balance. Stress-induced inflammation is another critical mechanism influencing hemostasis^[17]^. Stress activates both acute and chronic inflammatory responses, which have direct and indirect effects on the hemostatic system. Stress leads to the release of inflammatory cytokines such as IL-6, TNF-α, and C-reactive protein. These mediators promote a pro-inflammatory environment that supports clot formation and impairs the resolution of clotting. Chronic stress-induced inflammation can damage the endothelial lining of blood vessels. This endothelial dysfunction leads to increased expression of adhesion molecules, which facilitate platelet adhesion and activation, and enhances the procoagulant state. Inflammation affects both the coagulation and fibrinolytic systems. Inflammatory cytokines can increase the expression of TF, a key initiator of the coagulation cascade, and inhibit fibrinolysis, contributing to a hypercoagulable state.

Oxidative stress is another mechanism through which stress affects hemostasis. Stress-induced oxidative stress results from the imbalance between reactive oxygen species (ROS) and the body’s antioxidant defenses^[18]^. ROS can enhance platelet activation and aggregation. They promote the expression of activation markers on platelets and increase thromboxane A2 production, further contributing to clot formation. Oxidative stress can modify coagulation factors, increasing their activity. ROS can induce changes in the structure and function of clotting factors, enhancing their procoagulant properties. ROS can damage the vascular endothelium, leading to increased permeability and a procoagulant state. This damage disrupts the balance between procoagulant and anti-coagulant factors, promoting thrombus formation. Hormonal changes during stress also impact hemostasis. Besides catecholamines and cortisol, other hormones influenced by stress can affect blood clotting processes. Stress activates the Renin-Angiotensin-Aldosterone System leading to increased levels of angiotensin II and aldosterone^[19]^. Angiotensin II causes vasoconstriction and promotes platelet aggregation, while aldosterone affects fluid balance and blood pressure, which can influence hemostatic balance. In females, stress affects the levels of estrogen and progesterone, which have complex effects on hemostasis. Estrogen can increase clotting factor synthesis, while progesterone influences endothelial function and platelet aggregation.

Stress-induced hemostatic changes also involve interactions between the nervous system and endocrine responses^[20]^. Stress leads to the release of neurotransmitters such as serotonin, which can influence platelet function. Serotonin promotes platelet aggregation and the release of procoagulant factors. The neuroendocrine system regulates hemostasis through feedback mechanisms that balance stress responses with physiological processes. Dysregulation of these feedback systems can lead to sustained hemostatic changes and contribute to disease states. Genetic and epigenetic factors also play a role in how stress-induced hemostatic changes manifest^[21]^. Certain genetic polymorphisms can influence individual responses to stress and predispose individuals to stress-related hemostatic changes. For example, genetic variations in genes related to platelet function and coagulation can modulate the effects of stress on hemostasis. Stress can cause epigenetic changes that affect gene expression related to hemostasis. Epigenetic modifications such as DNA methylation and histone acetylation can influence the expression of genes involved in coagulation and inflammation. Psychological stressors can trigger the SNS and HPA axis responses, leading to changes in platelet function, coagulation, and fibrinolysis. This encompasses stressors such as intense exercise, trauma, or surgery. Physical stressors activate the body’s stress response systems, leading to acute changes in hemostasis including increased platelet activation, altered coagulation factor levels, and enhanced inflammatory responses.

### Psychological stress and hemostatic changes

Psychological stress, encompassing experiences such as anxiety, depression, and acute mental stress, has significant impacts on the hemostatic system^[22]^. The body’s response to psychological stress involves complex interactions between the nervous, endocrine, and immune systems, which collectively influence hemostasis. Psychological stress triggers the activation of the HPA axis and the SNS, leading to the release of stress hormones such as cortisol, adrenaline, and noradrenaline. These hormones have profound effects on hemostasis. As a primary glucocorticoid hormone, cortisol influences the synthesis and activity of various coagulation factors. Elevated cortisol levels can increase the production of procoagulant factors, such as fibrinogen and factor VIII, and decrease fibrinolytic activity, promoting a hypercoagulable state^[23]^. These catecholamines enhance platelet activation and aggregation by increasing the expression of adhesion molecules on platelet surfaces. This makes platelets more likely to clump together and form clots. Additionally, these hormones induce vasoconstriction, which can reduce blood flow and promote clot formation in small vessels. Numerous studies have documented the effects of psychological stress on hemostatic parameters^[^24,25^]^. For instance, individuals experiencing acute psychological stress, such as those undergoing mental arithmetic tasks or public speaking, show elevated levels of coagulation factors, including fibrinogen, factor VII, and D-dimer. These changes indicate an increased tendency for clot formation.

Chronic psychological stress, such as that associated with ongoing anxiety or depressive disorders, also leads to sustained alterations in hemostatic function. Research has shown that individuals with chronic stress exhibit persistently high levels of fibrinogen and other procoagulant factors^[26]^. This prolonged hypercoagulable state can contribute to the development and progression of atherosclerosis, a condition characterized by the buildup of plaques in the arterial walls, which can lead to cardiovascular events like myocardial infarction and stroke. Psychological stress activates the SNS, resulting in the release of adrenaline and noradrenaline. These hormones increase platelet reactivity and enhance the coagulation cascade, promoting clot formation. The release of cortisol during stress influences the production and activity of coagulation factors^[27]^. Cortisol can increase the synthesis of procoagulant factors and reduce fibrinolysis, the process that breaks down clots, leading to a pro-thrombotic state. Psychological stress induces the release of inflammatory cytokines, such as IL-6 and TNF-α^[28]^. These cytokines upregulate the production of coagulation factors and inhibit fibrinolysis, further promoting clot formation. The hemostatic changes induced by psychological stress have significant clinical implications^[29]^. Chronic stress is a well-established risk factor for cardiovascular diseases, including coronary artery disease and stroke. The sustained hypercoagulability associated with chronic stress can lead to the development of atherosclerotic plaques and increase the risk of thrombotic events.

### Physical stress and hemostasis

Physical stress, whether from intense exercise, trauma, or surgical procedures, significantly impacts the hemostatic system, which maintains the balance between blood clotting and fluidity^[30]^. This balance is crucial for preventing both excessive bleeding and pathological clot formation. The body’s response to physical stress involves a coordinated activation of the SNS and the release of stress hormones, such as adrenaline and noradrenaline. These hormones play a crucial role in preparing the body for physical exertion or injury by enhancing platelet reactivity and increasing the levels of procoagulant factors. Adrenaline and noradrenaline enhance the activation and aggregation of platelets^[31]^. This makes platelets more likely to clump together and form clots, a protective mechanism to prevent bleeding from potential injuries. Physical stress triggers the coagulation cascade, leading to the rapid formation of a stable blood clot^[32]^. The intrinsic and extrinsic pathways of the coagulation cascade are activated, resulting in the conversion of fibrinogen to fibrin, which stabilizes the platelet plug. Adrenaline and noradrenaline induce vasoconstriction, reducing blood flow to certain areas and promoting clot formation in small blood vessels. This helps minimize blood loss in case of injury. Regular, moderate exercise is generally beneficial for cardiovascular health. It improves endothelial function, enhances fibrinolytic activity, and helps maintain a healthy balance between coagulation and fibrinolysis. Moderate exercise has been shown to lower levels of fibrinogen and other procoagulant factors while increasing the activity of tPA, a key enzyme in breaking down clots^[33]^. Intense and prolonged physical activity can lead to a temporary hypercoagulable state. Studies have shown that immediately following intense exercise, there is an increase in platelet activation and elevated levels of coagulation factors, such as factor VIII and vWF. This heightened state of coagulation is thought to be an adaptive response to potential injuries but can increase the risk of thrombotic events, particularly in individuals with underlying cardiovascular risk factors^[^34,35^]^.

Severe physical trauma, such as from accidents or combat injuries, triggers a robust hemostatic response^[36]^. The body rapidly activates the coagulation cascade to form clots and prevent excessive blood loss. However, in cases of major trauma, this response can become dysregulated, leading to conditions such as DIC. DIC is characterized by widespread clotting and subsequent bleeding due to the consumption of clotting factors and platelets. Severe burns also induce significant hemostatic changes. The extensive tissue damage and inflammatory response associated with burns lead to the release of procoagulant factors and cytokines, which promote clot formation. At the same time, the extensive endothelial damage can impair the anticoagulant properties of the blood vessels, further tipping the balance toward a hypercoagulable state^[^37,38^].^ Surgical procedures, especially major surgeries, impose significant physical stress on the body, activating the hemostatic system. The surgical trauma leads to the release of TF, which triggers the extrinsic pathway of the coagulation cascade^[39]^. Additionally, the stress response to surgery includes the release of catecholamines and cortisol, further enhancing coagulation. After surgery, patients often experience a period of hypercoagulability, which increases the risk of VTE. Preventative measures, such as anticoagulant therapy and mechanical compression devices, are commonly used to mitigate this risk. Close monitoring and early mobilization are also important strategies to reduce the incidence of postsurgical thrombotic events.

The release of catecholamines (adrenaline and noradrenaline) enhances platelet function, increases the expression of procoagulant factors, and induces vasoconstriction^[40]^. Cortisol released during physical stress modulates the synthesis of coagulation factors and increases platelet sensitivity to activation, promoting clot formation. Physical stress induces an inflammatory response, leading to the release of cytokines such as IL-6 and TNF-α. These cytokines upregulate the production of coagulation factors and inhibit fibrinolysis, further promoting a hypercoagulable state. The hemostatic changes induced by physical stress have significant clinical implications, particularly in the management of patients undergoing surgery or those who have experienced trauma. In surgical patients, assessing the risk of thrombotic events and implementing appropriate prophylactic measures is essential. This includes the use of anticoagulants, mechanical compression devices, and early mobilization to reduce the risk of postoperative VTE. In trauma patients, rapid assessment and management of bleeding and coagulation status are critical. This may involve the use of blood products, antifibrinolytic agents, and other hemostatic interventions to stabilize the patient and prevent DIC^[40]^.

### Mechanisms of stress-induced hemostatic changes

Stress, whether psychological or physical, initiates a complex series of physiological responses that significantly influence the hemostatic system. This system is responsible for maintaining the balance between blood clot formation and dissolution, and stress can disrupt this balance, leading to either a hypercoagulable state (increased clotting) or a hypocoagulable state (increased bleeding). The SNS is a primary component of the body’s immediate response to stress. Activation of the SNS leads to the release of catecholamines, primarily adrenaline (epinephrine) and noradrenaline (norepinephrine), which have profound effects on the hemostatic system^[5]^. Adrenaline and noradrenaline enhance platelet reactivity by increasing the expression of surface adhesion molecules such as glycoprotein IIb/IIIa. This promotes platelet aggregation, making it easier for platelets to clump together and form clots. The increased platelet activation is crucial for rapid hemostasis in response to potential injury. Catecholamines induce vasoconstriction, narrowing blood vessels and reducing blood flow. This helps to limit blood loss in the event of injury but also creates conditions conducive to clot formation, particularly in small vessels.

The HPA axis is another critical pathway activated by stress. The hypothalamus releases corticotropin-releasing hormone, which stimulates the pituitary gland to secrete adrenocorticotropic hormone (ACTH). ACTH then prompts the adrenal cortex to produce cortisol, a glucocorticoid hormone with significant effects on hemostasis. Cortisol influences the synthesis and activity of several coagulation factors. It can increase the production of factors such as fibrinogen and factor VIII, enhancing the blood’s ability to clot. Cortisol also modulates the balance between procoagulant and anticoagulant factors, tipping the balance toward a procoagulant state during prolonged stress^[41]^. Cortisol has anti-inflammatory effects that can impact hemostasis. It reduces the production of certain inflammatory cytokines while modulating the activity of others. This can lead to changes in the endothelial cells lining blood vessels, affecting their anticoagulant properties and promoting clot formation.

Stress induces an inflammatory response, which plays a significant role in hemostatic changes^[42]^. The release of inflammatory cytokines such as IL-6, TNF-α, and interleukin-1 during stress can profoundly impact the coagulation system. IL-6 and TNF-α can upregulate the production of procoagulant factors and downregulate natural anticoagulants like protein C and antithrombin. This shift enhances the coagulation cascade and promotes clot formation. Inflammatory cytokines activate endothelial cells, which line the blood vessels. Activated endothelial cells express more TF, a key initiator of the extrinsic coagulation pathway. This activation further contributes to the hypercoagulable state observed during stress. Oxidative stress, characterized by an imbalance between the production of ROS and the body’s antioxidant defenses, is another mechanism through which stress impacts hemostasis. Elevated levels of ROS can damage endothelial cells, promote platelet activation, and enhance the coagulation cascade^[43]^. Oxidative stress can cause oxidative damage to endothelial cells, impairing their anticoagulant properties and increasing the expression of procoagulant factors such as TF and vWF. This promotes clot formation and reduces the ability of blood vessels to prevent unwanted clotting. ROS can directly activate platelets, increasing their aggregation and adhesion capabilities. This contributes to the hypercoagulable state often seen during periods of intense physical or psychological stress.

Various hormones released in response to stress, including cortisol, adrenaline, and noradrenaline, directly impact hemostatic processes^[20]^. The balance between these hormones and their interactions with other physiological systems is crucial in determining the net effect on hemostasis. Cortisol affects the synthesis and degradation of coagulation factors, promoting a shift toward a procoagulant state. Prolonged elevation of cortisol levels, as seen in chronic stress, can lead to sustained hypercoagulability. Adrenaline increases platelet reactivity and enhances the overall clotting potential of the blood. This effect is particularly pronounced during acute stress, where rapid clot formation can be lifesaving in the event of injury. The hemostatic changes induced by stress have significant implications for health, particularly in the context of cardiovascular diseases^[7]^. Chronic stress, with its sustained elevation of stress hormones and inflammatory mediators, can lead to persistent hypercoagulability, contributing to the development of atherosclerosis and increasing the risk of myocardial infarction and stroke. Acute stress episodes can precipitate thrombotic events, such as acute coronary syndrome, especially in individuals with preexisting cardiovascular conditions. The rapid increase in platelet activation and coagulation factors during acute stress can lead to the formation of occlusive clots, blocking blood flow to critical organs. Stress management techniques, such as mindfulness, cognitive-behavioral therapy, and physical exercise, can help reduce stress levels and improve hemostatic balance. Pharmacological interventions, including anticoagulants and anti-inflammatory agents, may also be beneficial in mitigating the risks associated with stress-induced hypercoagulability.

Stress-induced hemostatic changes have profound implications for health, influencing a range of conditions from cardiovascular diseases to autoimmune disorders. One of the most significant health implications of stress-induced hemostatic changes is the increased risk of cardiovascular diseases. Stress can lead to a state of chronic hypercoagulability, which contributes to the development and progression of atherosclerosis, a condition characterized by the buildup of plaques in the arterial walls^[44]^. Chronic stress results in elevated levels of procoagulant factors such as fibrinogen, factor VIII, and vWF. These factors promote the formation of atherosclerotic plaques and increase the risk of arterial blockages. Over time, these plaques can rupture, leading to acute cardiovascular events such as myocardial infarction (heart attack) or stroke. Acute stressors, such as intense psychological stress or severe physical exertion, can precipitate acute coronary syndromes. The immediate increase in platelet activation and coagulation factors can lead to the formation of thrombi in coronary arteries, causing angina or acute myocardial infarction. Managing stress through lifestyle changes, such as regular physical activity, a balanced diet, and relaxation techniques, can help reduce cardiovascular risk. Additionally, medications such as aspirin or statins may be prescribed to manage cardiovascular risk factors.

Physical and psychological stress can increase the risk of VTE, which includes deep vein thrombosis (DVT) and pulmonary embolism (PE)^[45]^. Stress-induced changes in hemostasis, such as increased platelet aggregation and clotting factor activation, contribute to the formation of blood clots in the veins. Stress can lead to a state of hypercoagulability that promotes clot formation in the deep veins of the legs. DVT can cause pain, swelling, and redness in the affected limb and, if left untreated, can lead to serious complications. A complication of DVT, PE occurs when a clot breaks loose and travels to the lungs, potentially causing symptoms like chest pain, shortness of breath, and even sudden death. Stress-induced hypercoagulability increases the risk of VTE events. To prevent and treat VTE, measures such as anticoagulant medications, mechanical compression devices, and early mobilization are recommended, especially in high-risk populations like postoperative patients. Stress-induced hemostatic changes also have implications for autoimmune disorders, where the immune system mistakenly attacks the body’s own tissues. Chronic stress can exacerbate autoimmune conditions through its effects on the inflammatory and hemostatic systems^[46]^. In conditions like systemic lupus erythematosus, stress can exacerbate symptoms by increasing inflammation and promoting a procoagulant state. Elevated levels of coagulation factors and inflammatory cytokines can worsen disease activity and increase the risk of thrombotic events. Stress can influence the progression of rheumatoid arthritis by modulating inflammatory responses and hemostatic factors. The increased production of pro-inflammatory cytokines and alterations in coagulation pathways can contribute to joint damage and disease progression. Effective stress management techniques, such as cognitive-behavioral therapy and stress reduction practices, can help manage autoimmune disorders. Additionally, medications that target inflammation and immune system activity may be used to control symptoms and disease progression.

Metabolic syndrome, a cluster of conditions including hypertension, obesity, insulin resistance, and dyslipidemia, is associated with an increased risk of cardiovascular diseases. Stress-induced hemostatic changes can exacerbate these conditions. Stress-induced SNS activation can lead to sustained high blood pressure, a key component of metabolic syndrome^[47]^. Chronic hypertension increases the risk of cardiovascular disease and stroke. Stress-related changes in cortisol levels can impair insulin sensitivity, contributing to the development of type 2 diabetes. Elevated cortisol levels are linked to increased blood glucose levels and insulin resistance. Lifestyle modifications, including regular exercise, a healthy diet, and weight management, are effective for managing metabolic syndrome. Additionally, medications to control blood pressure, glucose levels, and lipid profiles may be prescribed. Chronic stress can negatively impact cognitive function, including memory, attention, and executive functioning. Stress-induced hemostatic changes can exacerbate these effects through inflammatory and oxidative mechanisms^[48]^. Prolonged exposure to stress hormones like cortisol can impair memory formation and learning abilities. Stress-induced inflammation and oxidative stress can damage brain cells and disrupt cognitive processes. Chronic stress is a risk factor for mental health conditions such as anxiety and depression. The physiological changes associated with stress, including those affecting hemostasis, can contribute to the development and exacerbation of these conditions. Cognitive-behavioral therapy, mindfulness practices, and stress management techniques can help improve cognitive function and mental health. Additionally, pharmacological treatments for anxiety and depression may be prescribed as needed.

Stress-induced hemostatic changes during pregnancy can affect both maternal and fetal health^[49]^. Pregnancy itself is a procoagulant state, and stress can exacerbate this condition, leading to complications. Stress can contribute to the development of preeclampsia, a pregnancy complication characterized by high blood pressure and signs of damage to other organ systems. The condition involves increased coagulation and endothelial dysfunction. Maternal stress can impact fetal development and lead to adverse outcomes such as preterm birth or low birth weight. The effects of stress on maternal hemostasis can contribute to these risks. Stress reduction strategies for pregnant women, such as prenatal yoga, counseling, and adequate support, can help mitigate risks. Regular prenatal care and monitoring are also essential for managing stress-related complications during pregnancy. Stress-induced changes in hemostasis can affect the wound healing process^[50]^. While initial hemostasis is necessary for wound repair, excessive or prolonged stress can impair healing. Chronic stress can impair wound healing by disrupting inflammatory responses and increasing the risk of infection. Elevated cortisol levels and increased platelet activation can interfere with the repair processes. Effective stress management and proper wound care are essential for promoting optimal healing. Ensuring a balanced diet, adequate rest, and appropriate medical care can support the wound healing process^[^51–54^]^.

Stress can also impact gastrointestinal health through hemostatic and inflammatory pathways^[55]^. Stress can exacerbate gastrointestinal conditions, such as peptic ulcers, by increasing acid production and affecting mucosal protection. Stress-related hemostatic changes can also contribute to bleeding complications in these conditions. Stress can worsen symptoms of inflammatory bowel diseases such as Crohn’s disease and ulcerative colitis by modulating immune responses and inflammatory processes. Dietary modifications, stress reduction techniques, and medications to manage acid production and inflammation are important for maintaining gastrointestinal health. Stress-induced hemostatic changes can affect immune system function, potentially impacting the body’s ability to respond to infections and other challenges^[^56–60^]^. Chronic stress can suppress the immune system, making individuals more susceptible to infections and impairing the body’s ability to clear pathogens. Stress-induced inflammation can also contribute to immune system dysregulation. Stress management techniques, including relaxation exercises and adequate sleep, can help maintain immune system health. Vaccinations and preventive measures are also important for protecting against infections. Chronic stress affects overall well-being, influencing physical health, mental health, and quality of life. Chronic stress can lead to a range of health issues, reducing quality of life^[^61–65^]^. Effective management of stress through lifestyle changes, therapeutic interventions, and support systems is essential for improving overall well-being. Comprehensive stress management approaches, including psychological counseling, stress-reducing activities, and social support, can enhance quality of life and prevent stress-related health issues^[^66–69^]^.

## Conclusion

Stress-induced hemostatic changes represent a critical area of research with far-reaching implications for human health. Both psychological and physical stressors trigger complex physiological responses that alter hemostatic balance, potentially leading to a range of health issues from cardiovascular diseases to autoimmune disorders. The mechanisms underlying stress-induced hemostatic changes are multifaceted. Psychological stress activates the SNS and the HPA axis, leading to increased levels of catecholamines and cortisol. These hormones enhance platelet activation, promote coagulation factor synthesis, and induce a pro-inflammatory state, all of which contribute to a hypercoagulable condition. Physical stress, whether from intense exercise, trauma, or surgery, similarly impacts hemostasis through increased platelet aggregation, enhanced coagulation pathways, and inflammatory responses. Oxidative stress and hormonal influences also play significant roles in these processes, shaping how the body responds to both acute and chronic stressors.

The health implications of stress-induced hemostatic changes are profound and diverse. Chronic stress has been linked to cardiovascular diseases such as atherosclerosis, acute coronary syndromes, and VTE. Stress exacerbates these conditions through persistent activation of the coagulation system and inflammatory pathways. Additionally, stress affects autoimmune disorders, metabolic syndrome, cognitive function, and overall well-being, demonstrating its widespread impact on both physical and mental health. Effective management of stress is therefore crucial in preventing and treating these conditions, highlighting the need for comprehensive approaches that address both the physiological and psychological aspects of stress.

## Data Availability

Not applicable.
